# Radiological Evaluation of a Rare Case of Rib Caries and Cold Abscess in a Young Male Patient With Miliary Tuberculosis: A Case Report With Review of Literature

**DOI:** 10.7759/cureus.35075

**Published:** 2023-02-16

**Authors:** Prasanthi R Ghanta, Suresh V Phatak, Pratap S Parihar, Rajasbala P Dhande, Nidhi G Reddy

**Affiliations:** 1 Department of Radiodiagnosis, Jawaharlal Nehru Medical College, Datta Meghe Institute of Higher Education and Research, Wardha, IND; 2 Department of Radiodiagnosis, Narendra Kumar Prasadrao Salve Institute of Medical Sciences and Research Centre, Nagpur, IND

**Keywords:** rib caries, elastography, radiography, computed tomography, ultrasonography, miliary tuberculosis, cold abscess

## Abstract

Rib caries with a cold abscess is a rare presentation of tuberculosis (TB) and is tricky to diagnose. It is rarer in young patients, especially in conjunction with active miliary TB. We present one such case of a 23-year-old male patient who presented with swelling over the left lower chest. Rib caries and cold abscess were initially detected by ultrasonography and elastography. The rib involvement and the extent of the cold abscess were further evaluated on a computed tomography scan, which also showed active pulmonary miliary TB. The patient was treated by aspiration of the cold abscess and anti-tuberculosis therapy. The fact that the patient had no history of diabetes, alcoholism, human immunodeficiency virus infection, or immunodeficiency disorder increases the rarity of this case. This case highlights the role of imaging in diagnosing rib caries, cold abscess, and miliary TB.

## Introduction

Tuberculosis (TB) is one of the leading causes of mortality among infectious diseases globally and continues to have a high prevalence, especially in developing countries like India. The causative organism is the acid-fast bacillus *Mycobacterium tuberculosis*. Other mycobacteria can also cause respiratory and other illnesses; however, *M. tuberculosis* is the most predominant organism. *M. tuberculosis* most commonly affects the lungs, followed by lymph nodes, pleura, bones, joints, and the genitourinary system, and less commonly, causes miliary TB, TB meningitis, etc. [1]. The incidence of extrapulmonary TB increases with age and human immunodeficiency virus (HIV) co-infection [2]. Musculoskeletal TB is an uncommon form of TB, accounting for only 1-5% of TB cases, the most common of which is spinal TB/Pott’s spine, followed by TB involving the hip and knee joints. Cold abscesses involving the chest wall and rib caries are rare, constituting only about 0-5% of musculoskeletal TB and 0.1% of total TB cases. Only <50% of the patients who present with rib caries and cold abscesses have active pulmonary TB [3]. The diagnosis of rib caries is tricky because it is very uncommon and presents late because of its insidious onset. However, imaging plays a prominent role in its early diagnosis and evaluation of the extent of the disease. Diagnosis of rib caries can be confirmed by detecting the acid-fast tuberculous bacilli in the aspirate samples or caseating granulomas on histopathology.

## Case presentation

A 23-year-old male patient presented with a history of swelling over the left lower anterior chest region for four months. The swelling was initially small and gradually progressed in size with no associated pain. The patient also reported a history of cough with expectoration, weight loss of approximately 10 kg, loss of appetite, and evening rise of temperature associated with occasional chills and night sweats for six months. The cough and malaise aggravated in the two months preceding the presentation, and the increase in the size of the chest swelling caused concern for the patient. The patient was non-diabetic, non-smoker, and non-alcoholic. There was no history of hemoptysis or chest pain. The patient belonged to low socio-economic status. No family history of TB was reported. The patient was diagnosed with sputum smear-negative pulmonary TB and was prescribed anti-tuberculosis therapy (ATT) (rifampicin (5 mg/kg body weight) + isoniazid (10 mg/kg) + pyrazinamide (25 mg/kg) for two months of intensive phase followed by isoniazid and rifampicin for four months of continuation phase) five months back. He stopped taking ATT after three to four days of starting therapy and was lost to follow-up.

The general examination of the patient was unremarkable except for his lean body habitus (the body mass index (BMI) of the patient was 17 kg/m^2^, which made him underweight). The laboratory investigations of the patient including serum electrolytes, kidney and liver function tests, and urine examination were within normal limits. The complete blood count showed increased neutrophils in differential leukocyte count, peripheral smear revealed predominantly normocytic, normochromic red blood cells (RBCs) with few microcytes, and erythrocyte sedimentation rate (ESR) was raised. Enzyme-linked immunosorbent assay (ELISA) for HIV was negative (Table 1).

**Table 1:** Laboratory investigations. RBC = red blood cell; PS = peripheral smear; ESR = erythrocyte sedimentation rate; ELISA = enzyme-linked immunosorbent assay; HIV = human immunodeficiency virus; ref = laboratory values for normal reference range

Parameter	Result
Hemoglobin	14.4 g/dL (ref: 13.2–16.6 g/dL)
Total RBC count	5.57 million/mm^3^ (ref: 4.35–5.85 million/mm^3^)
Total leukocyte count	7,500 cells/mm3 (ref: 4,500–11,000 cells/mm^3^)
Neutrophils	70% (ref: 40–60%)
Lymphocytes	24% (ref: 20–40%)
Monocytes	4% (ref: 2–8%)
Eosinophils	2% (ref: 1–4%)
Basophils	0% (ref: 0.5–1%)
Platelet count	3.04 lakh/mm^3^ (ref: 1.5–4.5 lakh/mm^3^)
PS	Predominantly normocytic, normochromic RBCs with few microcytes
ESR	68 mm/hour (ref: 0–15 mm/hour)
ELISA for HIV	Negative

**Figure 1:**
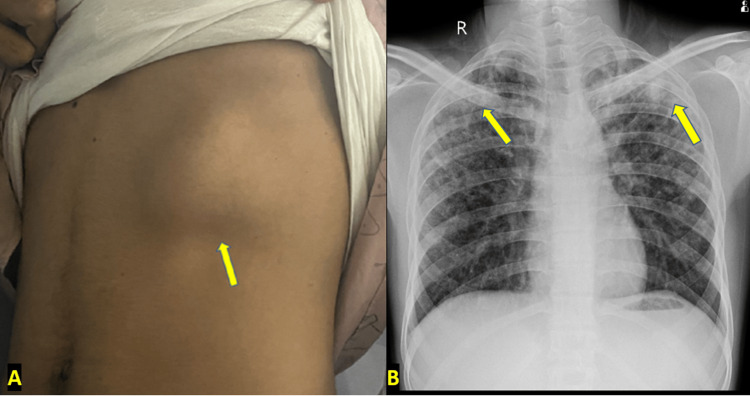
(A) Clinical image of the patient showing a swelling (depicted by arrow) over the left lower anterior chest region with no redness of the overlying skin. (B) Chest X-ray posteroanterior view of the patient showing alveolar opacities (depicted by arrows) and multiple miliary nodules in bilateral lung fields.

On local examination, a soft, non-tender swelling (Figure 1A) measuring approximately 7 cm × 5 cm was present over the left lower anterior chest, extending from the seventh to the ninth rib region. Swelling moved with inspiration and expiration, and the skin over the swelling was pinchable. The fluctuation test was positive. The swelling was non-reducible. No tenderness or local rise of temperature or redness of the overlying skin was present. Chest X-ray (posteroanterior view) showed alveolar shadows/opacities in bilateral upper zones and miliary shadows diffusely distributed throughout the bilateral lung fields (Figure 1B). No apparent mass was noted on radiography.

**Figure 2:**
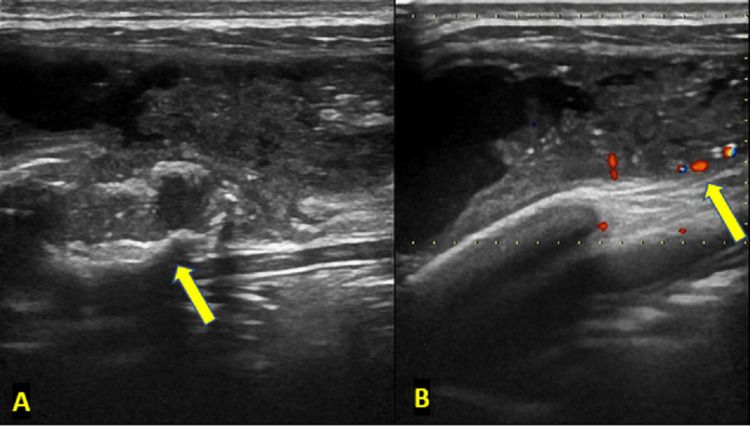
(A) B-mode gray scale ultrasound image showing rib erosion (depicted by arrow) with adjoining abscess cavity seen anterior to it. (B) Color Doppler image of the abscess showing minimal peripheral vascularity (depicted by arrow).

Ultrasonography (USG) of the swelling revealed a heterogeneous lesion (Figure 2A) with internal debris, tiny calcifications, and peripheral vascularity on Doppler (Figure 2B). There was associated erosion of the adjacent rib. No evidence of hepatosplenomegaly was noted.

**Figure 3:**
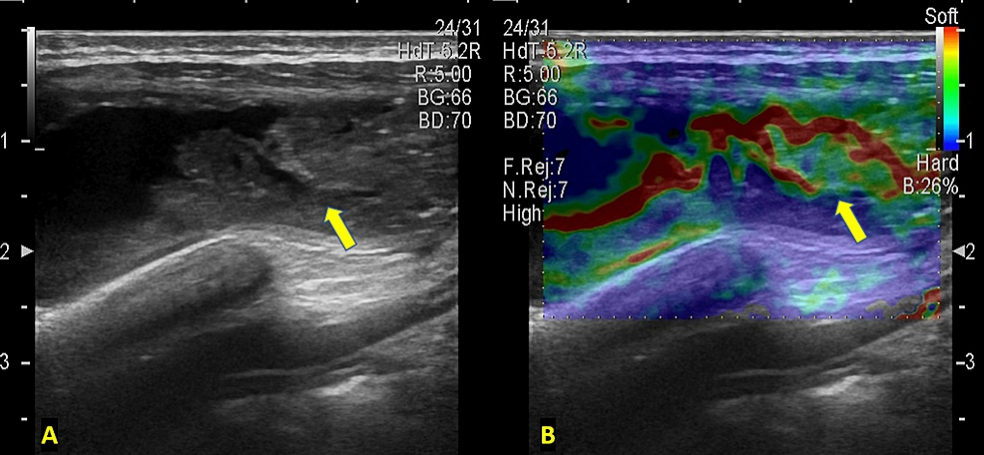
(A) B-mode ultrasound imaging showing a gray-scale image of the abscess (depicted by arrow) on the left. (B) The elastography image overlapped on the gray-scale image showing soft colors (depicted by arrow) red, yellow, and green with specks of aqua indicating softer consistency.

Elastography of the swelling (Figures 3A, 3B) showed soft colors (red, yellow, and green with specks of aqua) in the core of the lesion, indicating its softer consistency, surrounded by a periphery of blue color which showed the hard induration. Elastography also showed a longer extent of the soft core of the lesion (yellow arrow in Figure 3B) and delineated it from the similar-looking induration on the B-mode USG image. A preliminary diagnosis of rib caries with an adjacent infective collection was made, and the patient was referred for a computed tomography (CT) scan for further evaluation.

**Figure 4:**
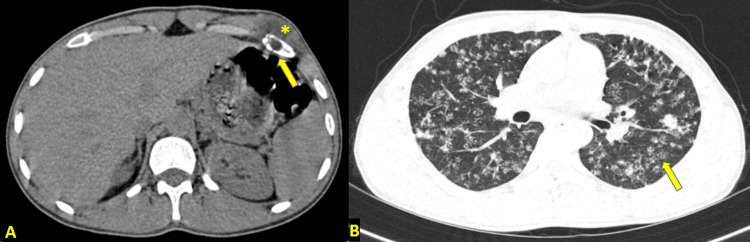
(A) HRCT image of the chest: soft-tissue window showing hypodense collection (marked by an asterisk) in the intramuscular plane of left external oblique muscle and eroded anterior part of the left seventh rib (depicted by arrow). (B) HRCT image of the chest: lung window showing multiple randomly distributed soft-tissue density nodules (depicted by arrow) diffusely distributed in bilateral lung fields, many of them showing a tree-in-bud pattern. HRCT = high-resolution computed tomography

On high-resolution computed tomography (HRCT) scan of the chest, a well-defined, thin-walled hypodense lesion/collection measuring 7.1 cm × 5.2 cm × 2.5 cm was noted involving the left external oblique muscle, anterior to the left seventh rib with associated destruction of the rib (Figure 4A showing breaks in both outer and inner cortex of the rib). The lesion extended anteriorly up to the subcutaneous plane and had no intraperitoneal extension posteriorly. The bilateral lung fields showed multiple randomly distributed miliary nodules with a tree-in-bud appearance and interlobular septal thickening in some places (Figure 4B). Few larger nodules were noted in bilateral upper lobes. No evidence of pleural effusion or mediastinal lymphadenopathy was present. The diagnosis was active miliary TB and rib caries with associated cold abscess.

USG-guided aspiration of the rib caries with cold abscess was performed, and samples from the aspirate and sputum were sent for microscopy, culture, and cartridge-based nucleic acid amplification test (CBNAAT) for *M. tuberculosis*. Microscopy and culture of the sputum sample did not reveal any tuberculous bacilli; however, CBNAAT of pus from the cold abscess was positive. The sputum smear continued to be negative throughout the course of the presentation and therapy. Cytology of the aspirate revealed a few epithelioid granulomas, lymphocytes, macrophages, and Langhan’s giant cells in a background of necrotic material.

The patient was advised to start ATT under direct observation (directly observed treatment, short course) for eight months in view of bony involvement and the previous lost to follow-up status of the patient. Treatment consisted of an intensive phase of three months of rifampicin (R) + isoniazid (H) + pyrazinamide (Z) + ethambutol (E) and streptomycin (S) for two months and H + R + Z + E for one month, followed by a continuation phase of five months of H + R + E. Weekly three doses were given under observation. The dosage of the drugs was 10 mg/kg body weight for isoniazid and rifampicin, 35 mg/kg for pyrazinamide, 30 mg/kg for ethambutol, and 25 mg/kg for streptomycin. Drug sensitivity testing of cultures grown from the aspirate showed no resistance to the first-line anti-tuberculosis drugs. The cold abscess showed good response to drainage and ATT with significant resolution at follow-up visits. The patient’s symptoms and chest X-ray also showed significant improvement at two months and complete resolution before the end of therapy.

## Discussion

The incidence of miliary and extrapulmonary TB has been on the rise due to HIV infections, including in developed countries [4]. Extrapulmonary TB involving the chest wall is very uncommon, and rib caries is even rarer [3].

Miliary or disseminated pulmonary TB can occur because of hematogenous or lymphatic spread from a tubercular focus [5]. Miliary TB is difficult to diagnose because of non-specific symptoms and atypical presentations. Diagnosis involves clinical, radiological, microbiological, or pathological criteria (Table 2).

**Table 2:** Criteria for diagnosis of miliary TB. TB = tuberculosis; ATT = anti-tuberculosis therapy; HRCT = high-resolution computed tomography

Serial number	Criteria for diagnosis
1	Clinical presentation of TB showing response to ATT
2	Typical pattern of miliary shadows on chest X-ray
3	Reticulonodular lung lesions that are bilateral and diffusely distributed on a background of a typical miliary pattern of shadows, demonstrable on chest X-ray or HRCT
4	Bacteriological/histopathological confirmation of TB

Miliary nodules are small (1-4 mm, commonly <2 mm in size) nodular opacities which are randomly and diffusely distributed in bilateral lung fields. They can show a reticulonodular pattern or a typical tree-in-bud appearance (tree-in-bud pattern depicts active infection) in some cases. Reticulonodular pattern with intra or interlobular septal thickening is caused by the distribution of miliary nodules along the septae or walls of alveoli. Radiography may only show hyperinflation in the initial phase of the disease and may take weeks to reveal a typical miliary pattern of opacities; hence, periodic radiographs in cases of high clinical suspicion can yield positive results. CT can demonstrate lymph node involvement, pleural involvement, and calcifications in addition to the miliary nodules and is also useful in miliary TB cases with atypical presentations which are difficult to diagnose on radiography. Another recently used modality is 18F-fluorodeoxyglucose positron emission tomography-computed tomography scan that can be used to evaluate the activity of known TB lesions, detect distant occult TB foci, guide biopsy procedures, and look for a response to ATT [6].

Chest wall TB can involve bones such as the vertebrae, costovertebral joints, ribs (rib shafts and costochondral junctions), and sternum. Chest wall cold abscesses can result from hematogenous spread from a primary pulmonary focus, rarely an extrapulmonary focus, or by direct extension from caseating lymph nodes. The caseating granulation tissue in the cold abscess that can cause pressure necrosis and the invading *M. tuberculosis* bacilli together result in erosion of the underlying rib or bone [7].

Rib caries with cold abscess presents late because of its insidious onset and lack of specific signs or symptoms. It can be confused with pyogenic abscess or benign or malignant lesions, or infectious conditions such as actinomycosis [8]. A cold abscess is differentiated from these lesions by imaging, and the confirmation is only by bacteriological or histopathological diagnosis. A cold abscess develops as a slowly growing swelling that may or may not be associated with pain. When associated with the destruction of the underlying bone or cartilage or pleuro-pulmonary tuberculosis, imaging is the mainstay of the preoperative evaluation.

CBNAAT has good sensitivity in tubercular cold abscesses, especially in aspirate samples containing pus [9]. Preoperative bacteriological confirmation from aspirates can be tricky as some cases are negative for tuberculous bacilli on microscopy and/or culture. In such cases, postsurgical histopathology of the excised abscess or the resected rib can show caseating granulomas [10]. However, in cases of high suspicion, such as the concurrent presence of pleuro-pulmonary tuberculosis and good response to ATT, preliminary diagnosis can be made with higher confidence with the help of imaging.

Radiography can be insensitive in chest wall TB until bony destruction is extensive, and chest wall involvement is not apparent until a mass (cold abscess) develops [3]. However, USG is a relatively widely available imaging modality that can be useful in diagnosing rib caries and cold abscesses [10]. B-mode USG imaging easily identifies the abscesses. Abscesses can be isoechoic, hypoechoic, or hyperechoic and are often heterogeneous on USG. Elastography helps identify isoechoic abscesses that are difficult to delineate on B-mode USG imaging. Elastography imaging shows the stiffness characteristics of tissues. The soft center of the abscess cavity shows a combination of softer colors, with red representing softest, yellow representing soft, and green representing medium stiffness. The surrounding induration shows harder colors, such as blue and dark blue. Thus, elastography helps differentiate the softer core of the abscess from the surrounding induration and the abscess from the surrounding normal tissue [11].

CT is necessary to evaluate chest wall tuberculosis to examine the extent of cold abscess, bony involvement, and any intrapleural extension of the lesion [7]. CT is particularly useful for evaluating the extent of bony destruction and sclerosis, whereas USG and magnetic resonance imaging (MRI) are better at delineating soft-tissue involvement in chest wall infections. USG and CT can also be used to guide image-guided percutaneous drainage and biopsy procedures, whereas MRI is used in extensive chest wall infections to guide preoperative planning [12,13]. However, MRI was not found to have any added diagnostic value in cold abscesses, according to a study by Boruah et al. [14].

Prompt imaging with USG and CT and biopsy procedures can lead to earlier diagnosis and favorable outcomes in cases of rib caries [15]. Surgical excision of the tubercular abscess with concomitant resection of the involved bone was the preferred approach of management because it has a high success rate, and ATT should be added to prevent a recurrence, according to a study by Keum et al. [10].

Cases similar to the present case with miliary pulmonary TB and isolated extrathoracic involvement of a single rib are rare in literature. Lee et al. conducted a single institution-based study on 54 cases of rib caries and reported male gender predilection and the most common age group of presentation as the third decade. The most common presenting symptom was swelling, followed by pain, pus discharge, and formation of a sinus, with an average symptom duration of one to six months at presentation; however, the duration ranged from five days to three years. Right hemithorax involvement was more common compared to the left. The most common lesion was an osteolytic lesion involving the shaft of a single rib, similar to the present case. Concomitant pulmonary TB was present in 40% of the cases most of which were inactive or minimally active disease, in contrast to the present case with active endobronchial spread. No other bone, joint, or spine involvement was reported, similar to our case [16].

Chang et al. reported a case of recalcitrant rib caries that presented as multiple chest wall lesions in a case of concomitant pulmonary TB with patchy subpleural consolidation. Lack of significant improvement two months after starting ATT with the H + R + Z + E regimen led to thoracotomy that showed caseous granuloma on a biopsy of the involved right fourth costal cartilage. One year later, new chest wall soft masses developed which led to a change in the ATT regimen to H + Z + ofloxacin + tuberactin and a second thoracotomy with resection of the involved parts of 10th and 11th ribs. Follow-up chest CT after four months showed significant resolution following the change in the ATT regimen [17]. In contrast, the present case had miliary tuberculosis and showed a good response to first-line ATT.

In a study by Irfan et al. on drug susceptibility in miliary TB cases, only 32 of the 110 cases with miliary TB were culture positive, and, of these, only three cases showed resistance to isoniazid. All culture-positive cases were sensitive to rifampicin, ethambutol, pyrazinamide, and streptomycin. Hence, they concluded that drug-sensitive mycobacteria continue to be implicated in the causation of miliary TB and that initial empirical therapy can be given with first-line anti-TB drugs [18].

In a retrospective comparative study on miliary TB by Kim et al., which included 15 patients with HIV-seropositive status and 14 patients with HIV-seronegative status, statistical analysis revealed significant differences in the radiological findings in the form of lower prevalence of larger nodules and higher prevalence of extrathoracic involvement, necrotic lymph nodes, and interlobular septal thickening in HIV-seropositive cases [19]. In the current case with HIV-seronegative status, some interlobular septal thickening was seen; however, there was no lymphadenopathy and pleural involvement. Extrathoracic involvement in the form of concomitant rib caries and cold abscess were seen.

Murthy et al. reported a case of rib caries in an 18-year-old lady who presented with a non-tender swelling over the back in the left infra-scapular region measuring 10 cm × 10 cm. No local inflammatory signs were present. Her laboratory investigations revealed mild anemia and raised ESR. Chest X-ray showed blunting of the left costophrenic angle and opacity in the left lower zone. CT confirmed the presence of pleural effusion, features of pulmonary TB in the left lower lobe, destruction of the left sixth rib, and the presence of the left posterior chest wall cold abscess. There was also cervical and mediastinal lymphadenopathy. Fine-needle aspiration cytology of the cold abscess and right cervical lymph node revealed acid-fast bacilli (AFB). A sputum smear was also positive for AFB. The patient had good recovery with drainage of the cold abscess and ATT [7]. Though the presentation and treatment of the cold abscess were similar, the patient did not have miliary pulmonary TB but had concomitant pulmonary TB, as well as neck and mediastinal tubercular lymphadenitis, in contrast to the present case.

Ilyas et al. reported an interesting case of disseminated miliary TB which presented with numerous non-specific symptoms due to the involvement of multiple organs. The diagnosis was unfortunately made posthumously when the blood and stool samples returned positive for *M. tuberculosis* complex in addition to previously detected *M. avium *complex. They highlighted the diagnostic challenge posed by *M. tuberculosis* due to its nature of being a great imitator of numerous other diseases [20]. Hence, prompt diagnosis and treatment can make a difference between life and death in this age-old disease plaguing humankind.

## Conclusions

Rib caries with a cold abscess involving the chest wall is very rare in a case of active pulmonary TB. Radiography is initially insensitive in detecting miliary TB and cold abscesses. CT is essential in diagnosing miliary TB, especially in atypical presentations, and in rib caries to delineate sclerosis and erosion of bones and complications such as intrapleural extension; however, USG is easily available and beneficial in detecting abscesses and rib erosion. Diagnosis is confirmed by the detection of *M. tuberculosis* bacilli or on histopathology by demonstrating caseating granulomas.

In this case, though radiography could not initially detect the cold abscess, it was useful in showing the alveolar shadows and miliary nodules. USG was able to detect the abscess cavity and rib erosion. Elastography was useful in demarcating the extent of the abscess cavity and the periphery of induration. CT further showed the extent of chest wall involvement (showed cortical breaks in the rib and demarcated the extent of the cold abscess) and ruled out intrapleural or intraperitoneal extension of the cold abscess. HRCT of the chest was also useful in showing the diffusely distributed miliary nodules with a tree-in-bud pattern and interlobular septal thickening, thus diagnosing the concurrent presence of active miliary TB in the patient. The diagnosis was confirmed by the detection of *M. tuberculosis* on CBNAAT and the patient responded well to aspiration of the abscess and ATT. This case report demonstrates the typical radiological findings of rib caries and miliary TB on different imaging modalities.
